# Conditional belonging under institutional ambiguity: narratives of Sudanese asylum seekers in Eilat, Israel

**DOI:** 10.3389/fsoc.2026.1943355

**Published:** 2026-10-12

**Authors:** Keren Beja Weinberg, Miguel A. Maldonado, Manuel Moyano

**Affiliations:** 1 Departamento de Psicología, Facultad de Ciencias de la Educación y Psicología, Universidad de Córdoba, Córdoba, Spain; 2 Hospital Universitario “Reina Sofía”, Instituto Maimónides de Investigación Biomédica de Córdoba (IMIBIC), Córdoba, Spain

**Keywords:** forced migration, asylum seekers, Sudan, Israel, social integration, community support, institutional ambiguity, conditional belonging

## Abstract

**Introduction:**

This qualitative study explores how Sudanese asylum seekers living in Eilat, Israel, narrate belonging under conditions of prolonged legal and institutional ambiguity.

**Methods:**

Based on semi-structured in-depth interviews with 30 participants, the study examines everyday experiences across employment, education, welfare and health services, and housing, while remaining open to emergent themes, including the role of the Sudanese community. The analysis combines a multidimensional integration framework with intergroup contact theory to assess how participation in key domains interacts with status relations and institutional support.

**Results:**

The analysis identified five interrelated themes: traumatic rupture and forced displacement; institutional rejection upon arrival; Eilat as a space of partial reconstruction; conditional belonging; and the dual protective and constraining role of the Sudanese community. Across employment, housing, education, and welfare and health, participants described functional integration and everyday stabilisation without corresponding legal security or broader social inclusion. Contact with members of the host society was described as largely instrumental, whereas co-ethnic networks provided essential material and emotional support in a context where opportunities for bridging ties remained limited.

**Discussion:**

The study contributes to contemporary debates on conditional belonging by offering an empirically grounded account from a peripheral city in which labour demand coexists with constrained civic inclusion.

## 1 Introduction

Forced migration, particularly involving refugees and asylum seekers, constitutes one of the central humanitarian, political, and social challenges confronting contemporary societies (Castles et al., 2013). By the end of 2024, an estimated 123.2 million people worldwide had been forcibly displaced (United Nations High Commissioner for Refugees, 2025a). In Israel, 26,151 asylum seekers and 603 recognized refugees were registered as of 31 December 2024 (United Nations High Commissioner for Refugees, 2025b). Despite its historical identity as a state established in the aftermath of persecution and displacement, Israel has not developed a consistent long-term policy governing the legal status and long-term integration of asylum seekers. Instead, asylum policy has been characterized by prolonged institutional ambiguity, leaving asylum seekers protected from deportation but without a stable legal status or a clear pathway to long-term social integration (Amran, 2014; State Comptroller and Ombudsman of Israel, 2018).

Since the mid-2000s, Israel has received asylum seekers, primarily from Sudan and Eritrea, without developing a coherent policy governing their legal status and social rights. Consequently, many asylum seekers have remained without full access to basic social protections and have relied extensively on civil society organisations and non-governmental organisations for legal, humanitarian, and social assistance (ASSAF, 2020; Hotline for Refugees and Migrants, 2021; State Comptroller and Ombudsman of Israel, 2018). This prolonged institutional uncertainty has reinforced insecurity, fostered dependence on non-governmental support, and limited opportunities for long-term integration into Israeli society (Amran, 2014; Donato and Ferris, 2020; Hynie, 2018).

Against this background, the city of Eilat represents an analytically distinctive case study. As a peripheral border city whose economy relies heavily on tourism and hospitality, Eilat has experienced persistent labour shortages that have created a longstanding dependence on labour recruited from outside the city, including foreign workers. This reliance on labour recruited from outside the city predates the arrival of African asylum seekers. Eilat’s transformation into a large-scale tourism destination accelerated during the 1980s, accompanied by substantial hotel development and growing demand for a mobile workforce (Benner, 2023). In 1988, legislation extending financial incentives to discharged Israeli soldiers who took temporary employment in hotels contributed to an influx of young workers into Eilat, illustrating the city’s longstanding dependence on workers recruited from outside the local labour market (Belhassen, 2012; Benner, 2023). Thus, when African asylum seekers began arriving through the Egyptian border in the mid-2000s, they entered a local economy whose tourism sector had already developed a structural reliance on externally recruited labour. Within the broader Israeli labour market, this pattern developed alongside policies that increasingly relied on temporary migrant labour in low-skilled sectors and on employment arrangements characterised by prolonged temporariness and limited social protections (Kemp, 2010). During the 2000s, as African asylum seekers began entering Israel through the Egyptian border, Eilat became one of the urban locations in which they were able to establish relatively stable employment and housing. Yacobi (2011), for example, documented approximately 1,200 African asylum seekers in Eilat at the time of his study, mainly from Southern Sudan, many of whom were employed in the city’s tourist resorts; he described Eilat as a setting in which access to work and housing distinguished the local experience from the more precarious conditions documented elsewhere in Israel. These developments indicate that the establishment of a Sudanese asylum-seeking community in Eilat was not incidental but was closely connected to the structural characteristics of the city’s tourism-based labour market and its continuing demand for workers. This pattern is also consistent with more recent research highlighting the importance of Eilat’s hospitality sector in shaping the economic incorporation of asylum seekers in the city (Amran, 2014; Barak-Bianco and Raijman, 2025). Although Eilat has thus become an important employment hub for asylum seekers in Israel, it has received remarkably limited scholarly attention compared with Tel Aviv, where much of the existing research has focused on asylum seekers’ experiences and their interactions with municipal authorities, professionals, and civil society organisations (Lev Ari and Herscovici, 2025; Herscovici and Lev Ari, 2025). This distinctive historical and local context provides a valuable opportunity to examine how processes of integration and belonging evolve under conditions of prolonged institutional ambiguity. While employment may create opportunities for economic participation and everyday stability, whether such participation also develops into broader social inclusion and secure belonging remains an open empirical question.

Despite the growing body of research on asylum seekers in Israel, relatively little is known about how integration and belonging develop in peripheral urban settings such as Eilat, where labour-market participation, geographical location, and everyday contact with the host population create a context that differs substantially from that of Tel Aviv. Addressing this gap, the present study explores the lived experiences of Sudanese asylum seekers in Eilat and examines how institutional conditions shape processes of integration and belonging across four central domains of everyday life: employment, education, housing, and welfare and health.

Drawing on semi-structured in-depth interviews with 30 Sudanese asylum seekers residing in Eilat, this study seeks to contribute to the literature on forced migration by providing an empirically grounded understanding of how prolonged institutional ambiguity shapes everyday experiences of integration and belonging within an under-researched urban context.

## 2 Theoretical framework

This study seeks to understand how processes of integration and belonging are shaped among Sudanese asylum seekers living in Eilat under conditions of prolonged institutional ambiguity. Addressing this question requires a theoretical framework that explains both the structural conditions that facilitate integration and the social mechanisms through which everyday interactions may or may not develop into a sense of belonging. Accordingly, this study draws on two complementary theoretical perspectives: Ager and Strang’s (2008) model of integration domains and Allport’s (1954) intergroup contact theory. Together, these frameworks provide an integrated analytical lens for examining not only whether opportunities for integration exist, but also how institutional conditions influence the extent to which such opportunities foster meaningful social inclusion and belonging.

### 2.1 Ager and Strang’s model of integration

Ager and Strang's (2008) model is widely regarded as one of the most influential theoretical frameworks for understanding the integration of refugees and asylum seekers in host societies. Rather than conceptualising integration as a fixed outcome, the model views it as a multidimensional process shaped by the interaction of several structural and social domains.

The framework identifies four core domains of integration, employment, education, housing, and health, alongside key facilitators, including safety and stability, language and cultural knowledge, and forms of social connection, namely social bonds, social bridges, and social links. These domains do not guarantee successful integration; rather, they create the structural conditions and opportunities for participation in everyday life and for interaction with the host society (Ager and Strang, 2008).

While Ager and Strang (2008) identify health as one of the core integration domains, the present study examines welfare and health together. This adaptation reflects the Israeli context, where access to healthcare is closely intertwined with access to welfare services and where both domains are shaped by the same institutional constraints affecting asylum seekers.

In the present study, Ager and Strang’s model provides the organisational framework for analysing the four domains examined: employment, education, housing, and welfare and health. It enables an assessment of how structural conditions facilitate or constrain integration while distinguishing between functional participation in the institutions of the host society and the development of a broader sense of social belonging.

### 2.2 Allport’s intergroup contact theory

Allport's (1954) intergroup contact theory provides an explanatory lens for examining how participation in everyday social settings may be associated with changes in intergroup attitudes and relations. The theory does not treat contact itself as sufficient; rather, it directs attention to the conditions under which contact is most likely to produce positive intergroup outcomes. According to Allport (1954), these conditions are equal status within the contact situation, common goals, intergroup cooperation, and support from authorities, law, or custom. Subsequent evidence indicates that these conditions strengthen the association between contact and reduced prejudice but are facilitative rather than strictly necessary (Pettigrew and Tropp, 2006).

Subsequent research has expanded this framework by demonstrating that the outcomes of intergroup contact are also shaped by broader psychosocial and structural factors, including group identification, intergroup expectations, intergroup anxiety, and the wider social and institutional context in which contact occurs (Brown et al., 1999; Pettigrew and Tropp, 2006; Vorauer and Kumhyr, 2001). These factors may reinforce intergroup boundaries even when everyday contact exists, helping to explain why participation in domains such as employment, education, housing, and welfare and health does not necessarily lead to broader social inclusion.

In the present study, Allport’s theory complements Ager and Strang’s framework by directing attention to the quality of contact between Sudanese asylum seekers and members of the host society across the four domains examined. The four conditions are used as sensitising criteria for assessing whether recurring interactions remain role-bound and instrumental or develop into more reciprocal cross-group relationships. Any connection between contact and belonging is therefore treated as an empirical question rather than assumed in advance.

### 2.3 An integrated analytical framework

Ager and Strang's (2008) model and Allport's (1954) intergroup contact theory offer complementary perspectives. Whereas Ager and Strang identify the structural domains and social connections through which integration is organised, Allport directs attention to the conditions under which intergroup contact within those domains is associated with more positive intergroup outcomes. Allport’s theory explains the social conditions under which such participation may develop into meaningful social integration and a sense of belonging.

Combining these two frameworks makes it possible to examine not only whether opportunities for integration exist, but also why functional participation does not necessarily translate into broader social inclusion. In other words, the study distinguishes between participation in the institutions of the host society and the quality of the social relationships that emerge within these settings, examining how institutional conditions facilitate or constrain the transition from participation to belonging.

This integrated analytical framework is applied to the experiences of Sudanese asylum seekers across four central domains of everyday life, employment, education, housing, and welfare and health. It provides a comprehensive lens for analysing how prolonged institutional ambiguity shapes both the structural conditions for integration and the quality of intergroup contact, thereby influencing opportunities for meaningful social inclusion and the development of a sense of belonging within the unique urban context of Eilat.

Within this integrated framework, two related concepts are used to interpret the empirical material: institutional ambiguity and conditional belonging. Institutional ambiguity refers to the prolonged discrepancy between sustained participation in the social and economic life of the host society and the absence of stable institutional recognition, legal security, and predictable long-term membership. This condition is particularly relevant to asylum seekers whose prolonged residence and participation coexist with a liminal legal status and restricted access to social rights (Barak-Bianco and Raijman, 2025). Belonging, in turn, is understood as a relational and socially situated process through which individuals develop attachments, recognition, and a sense of membership within particular social and institutional contexts (Yuval-Davis, 2006). Building on these perspectives, the term conditional belonging is used in this study as an analytical concept to describe situations in which individuals become socially, economically, and emotionally rooted in a place while the security and recognition of that belonging remain contingent upon institutional conditions beyond their control. Conditional belonging is therefore not introduced as an additional theoretical framework, but as an empirically grounded concept interpreted through the combined analytical lens of Ager and Strang’s integration framework and Allport’s intergroup contact theory.

## 3 Literature review

### 3.1 Employment

Employment is widely recognised as a central domain of refugee integration, providing opportunities for economic participation, social interaction, and long-term inclusion within the host society (Ager and Strang, 2008). However, participation in the labour market alone does not guarantee broader social integration, as its outcomes depend on institutional conditions, employment quality, and opportunities for meaningful interaction with members of the host society.

In Israel, the employment of asylum seekers, particularly those from Sudan and Eritrea, is characterised by prolonged legal ambiguity and the absence of a comprehensive regulatory framework governing their legal status and labour rights. Their temporary legal status creates persistent uncertainty for both workers and employers, contributing to precarious labour-market participation and long-term economic insecurity (Amran, 2014; State Comptroller and Ombudsman of Israel, 2018; Barak-Bianco and Raijman, 2025).

Structural barriers, including the limited recognition of educational qualifications, restrictions associated with temporary legal status, and limited access to employment-related social protections, frequently confine asylum seekers to low-paid positions that do not reflect their education, professional experience, or skills. These constraints contribute to persistent underemployment, restricted occupational mobility, and limited opportunities for long-term economic advancement (Barak-Bianco and Raijman, 2025; State Comptroller and Ombudsman of Israel, 2018).

The Deposit Law further reinforced these structural constraints by requiring a proportion of asylum seekers’ wages to be deposited in a designated fund that became accessible only upon departure from Israel. Although subsequent amendments reduced the financial burden for some groups, many asylum seekers continued to experience restricted access to employment-related rights and social protections, reinforcing the insecurity associated with their temporary legal status (State Comptroller and Ombudsman of Israel, 2018; Barak-Bianco and Raijman, 2025).

Employment conditions are particularly challenging for women, who face additional gender-based vulnerabilities, including discrimination during pregnancy, dismissal, and limited access to maternity-related rights and benefits (Slonim-Nevo and Lavie-Ajayi, 2017; State Comptroller and Ombudsman of Israel, 2014).

From the perspective of Allport’s (1954) intergroup contact theory, workplaces may provide valuable opportunities for sustained interaction between asylum seekers and members of the host society. However, where equal status, institutional support, shared goals, and cooperation are limited, the presence of repeated workplace contact alone provides insufficient grounds for inferring broader social inclusion (Allport, 1954; Pettigrew and Tropp, 2006). Consequently, employment may facilitate economic participation without necessarily fostering a stronger sense of belonging. Examining both the structural conditions of employment and the quality of workplace interactions is therefore essential for understanding how employment contributes to, or constrains, the integration of Sudanese asylum seekers in Israel.

### 3.2 Education

Education is widely recognised as one of the central domains of refugee integration, contributing not only to academic achievement but also to language acquisition, social participation, and the development of a sense of belonging (Ager and Strang, 2008). The extent to which education promotes integration, however, depends on institutional facilitators, including language support, cultural mediation, safety, stability, and meaningful connections between schools, families, and the wider community.

In Israel, children of asylum seekers experience multidimensional vulnerability shaped by the interaction of legal uncertainty, educational disadvantage, and psychosocial stressors (ASSAF, 2023; Hotline for Refugees and Migrants, 2021; Rozen et al., 2022). Although Israel is a signatory to the United Nations Convention on the Rights of the Child, guaranteeing every child the right to education, health, and an adequate standard of living (United Nations, 1989), the realization of these rights remains only partial. While the Ministry of Education formally recognises refugee children’s right to education under the Compulsory Education Law of 1949, many continue to experience limited access to tailored educational support, language mediation, and culturally responsive services (ASSAF, 2023).

These challenges begin in early childhood. In the absence of formal frameworks for infants and toddlers, many children are placed in unregulated childcare settings characterised by overcrowding, inadequate supervision, limited developmental stimulation, and poor living conditions (ASSAF, 2023; Hotline for Refugees and Migrants, 2021; Rozen et al., 2022). As they enter preschool, prolonged exposure to poverty, instability, and restricted access to early childhood services often results in substantial linguistic, emotional, and developmental gaps that affect school readiness and subsequent educational integration (ASSAF, 2023; Rozen et al., 2022).

These educational inequalities continue throughout the school years. Many refugee children are classified as being at risk and grow up in environments characterised by poverty, neglect, violence, and limited access to basic resources (ASSAF, 2023). Teachers frequently encounter significant gaps in school readiness, language development, classroom participation, attention, and socio-emotional functioning. Nutritional and health-related difficulties further impair children’s daily functioning and learning capacity, while the absence of linguistic and cultural mediation weakens communication between schools and families and reduces children’s ability to fully realise their educational rights (ASSAF, 2023; Rozen et al., 2022).

These vulnerabilities often intensify during adolescence. Upon reaching adulthood, many young asylum seekers lose the limited protections available during childhood while continuing to live under temporary legal status. Restricted access to higher education, stable employment, and broader social opportunities reinforces uncertainty regarding their future and limits upward social mobility (ASSAF, 2023; Hotline for Refugees and Migrants, 2021; State Comptroller and Ombudsman of Israel, 2018). As one young woman quoted in ASSAF’s report stated, ‘I turned 18 and my life was stuck. I feel like I wasted 12 years of schooling’ (ASSAF, 2023, p. 32). This is not a quotation from a participant in the present study. These cumulative experiences contribute to educational disengagement, social marginalisation, and weakened belonging. Some young people even attempt to conceal their refugee identity by presenting themselves as members of the Ethiopian community in an effort to gain greater social acceptance (Rozen et al., 2022).

Comparative evidence from the European Union demonstrates a more comprehensive approach to the educational inclusion of refugee children. Member States are required to provide access to education within three months of an application for international protection (European Parliament and Council of the European Union, 2013). More recently, the European Commission (2022) has emphasised the importance of structured language programmes, cultural mediation, psychosocial support, and targeted educational measures to promote both educational achievement and social inclusion. By contrast, the Israeli context continues to be characterised by significant gaps in tailored educational support, particularly for children with special educational needs, together with limited parental involvement resulting from linguistic, cultural, and institutional barriers.

From the perspective of Allport's (1954) intergroup contact theory, schools represent one of the principal settings in which sustained interaction between children of asylum seekers, their families, and members of the host society can occur. However, positive outcomes depend on institutional support, opportunities for cooperation, relative equality of status, and culturally responsive educational practices, including language mediation and inclusive school environments. In the absence of these conditions, participation in education may not translate into broader social integration and a stronger sense of belonging. Examining education through both structural and relational perspectives therefore provides important insight into how educational experiences facilitate, or constrain, the integration of Sudanese asylum seekers in Israel.

### 3.3 Housing

Housing is widely recognised as one of the central domains of refugee integration, providing the stability and security necessary for participation in the host society. Beyond ensuring physical shelter, housing influences access to employment, education, welfare and health services, opportunities for social interaction, and the development of a sense of belonging (Ager and Strang, 2008; Hynie, 2018).

In Israel, the housing situation of asylum seekers reflects the absence of a coherent governmental policy designed to provide humanitarian assistance and secure basic social rights. Asylum seekers entering Israel through the Egyptian border were commonly released from detention facilities without structured housing arrangements, institutional guidance, or practical support, leaving many to secure accommodation independently under highly precarious conditions (State Comptroller and Ombudsman of Israel, 2014; Hotline for Refugees and Migrants, 2021).

Unlike European Union Member States, which are required to provide material reception conditions, including accommodation, for applicants for international protection (European Parliament and Council of the European Union, 2013), Israel has not established a comprehensive housing framework for asylum seekers. Consequently, many are compelled to rely on informal and overcrowded housing arrangements, primarily in socioeconomically disadvantaged neighbourhoods characterised by residential instability, inadequate infrastructure, and limited institutional support (ASSAF, 2023; Hotline for Refugees and Migrants, 2021; State Comptroller and Ombudsman of Israel, 2018).

Housing insecurity is reflected in overcrowded apartments, shared sleeping arrangements, unsafe electrical connections, and hazardous use of gas canisters, conditions that pose immediate physical risks while undermining personal security, well-being, and opportunities for long-term rehabilitation (ASSAF, 2020). These circumstances disproportionately affect women, children, and people with disabilities, many of whom experience homelessness, severe economic deprivation, and limited access to community or institutional support. Existing welfare services provide only partial assistance, leaving many asylum seekers without adequate therapeutic, rehabilitative, or long-term support and reinforcing processes of social exclusion (ASSAF, 2020; Hotline for Refugees and Migrants, 2021; Rozen et al., 2022; State Comptroller and Ombudsman of Israel, 2014; State Comptroller and Ombudsman of Israel, 2018).

Available assistance remains limited and is largely restricted to particularly vulnerable cases, leaving no comprehensive response to long-term housing insecurity (ASSAF, 2020; State Comptroller and Ombudsman of Israel, 2014). Consequently, the vast majority continue to rely on overcrowded informal housing arrangements that perpetuate instability and increase exposure to physical, social, and economic vulnerability (Rozen et al., 2022).

Against this background, examining housing conditions within the distinctive context of Eilat provides an opportunity to explore how prolonged institutional ambiguity, residential stability, and local labour-market conditions interact to shape refugees’ opportunities for integration and belonging.

From the perspective of Allport’s (1954) intergroup contact theory, residential environments may facilitate everyday interaction between asylum seekers and members of the host society. However, segregated and unstable housing patterns restrict equal-status encounters and reflect the absence of institutional support, two of the conditions identified by Allport as essential for positive intergroup contact. Consequently, housing insecurity limits opportunities for meaningful social relationships and prevents everyday contact from developing into broader social inclusion and a stronger sense of belonging. Residential stability therefore represents not only a material resource but also a fundamental condition for social participation, community continuity, and the development of belonging within the host society (Ager and Strang, 2008; Hynie, 2018; Yuval-Davis, 2006).

### 3.4 Welfare and health

Welfare and health are widely recognised as central domains of refugee integration, extending beyond access to medical care to encompass safety, well-being, psychosocial resilience, and the capacity to participate fully in the host society (Ager and Strang, 2008). Access to welfare and health services influences not only physical and mental well-being but also refugees’ opportunities to engage in education, employment, and community life, making these domains fundamental to long-term integration.

In Israel, the welfare and health of asylum seekers are characterised by persistent structural barriers, prolonged legal uncertainty, and limited institutional support (ASSAF, 2023; Hotline for Refugees and Migrants, 2021; State Comptroller and Ombudsman of Israel, 2018). Economic hardship, unresolved migration-related trauma, and restricted eligibility for public services compel many asylum seekers to prioritise immediate survival over long-term integration. Although the Supreme Court ruled in 2014 that urgent welfare services should be expanded for asylum seekers, implementation has remained limited, leaving many dependent on non-governmental organisations for essential welfare assistance (Hotline for Refugees and Migrants, 2021; State Comptroller and Ombudsman of Israel, 2018). These structural vulnerabilities became particularly evident during the COVID-19 pandemic, when approximately 80 per cent of asylum seekers lost their employment or were placed on unpaid leave, resulting in severe food insecurity, deteriorating health conditions, and heightened social vulnerability (Hotline for Refugees and Migrants, 2021).

Access to welfare services remains largely restricted to emergency interventions targeting particularly vulnerable groups, including survivors of violence, people experiencing homelessness, and individuals with disabilities. Preventive and community-based services remain limited, while long-term rehabilitation and sustained psychosocial support are largely unavailable. Consequently, many structural vulnerabilities remain unaddressed despite substantial evidence documenting their cumulative social and health consequences (ASSAF, 2020; ASSAF, 2023; Hotline for Refugees and Migrants, 2021; Rozen et al., 2022; State Comptroller and Ombudsman of Israel, 2018).

Access to healthcare is similarly constrained. Asylum seekers are generally excluded from Israel’s National Health Insurance Law and therefore have only limited access to publicly funded healthcare services, relying instead on emergency medical care, non-governmental organisations, and community-based providers (Kritzman-Amir, 2015). Children without legal status may be insured through a special arrangement with Meuhedet Health Services, which offers coverage similar in scope to that available to Israeli children. However, monthly premium payments and accumulated debt prevent some families from maintaining continuous coverage. Consequently, some children remain uninsured and experience barriers to timely medical care (ASSAF, 2023).

In response to these gaps, community organisations have become the principal providers of welfare and health services for asylum seekers. Organisations such as Mesila offer emotional support, paramedical services, legal assistance, and specialised programmes for survivors of trafficking and slavery (Tel Aviv–Yafo Municipality, n.d.). At the same time, repeated efforts by civil society organisations to promote policy reform through research, policy papers, and petitions to the High Court of Justice have achieved only limited implementation (ASSAF, 2020). This situation highlights the discrepancy between the widespread public perception of asylum seekers as a burden on Israel’s welfare and healthcare systems and the documented limitations in their actual access to these services (ASSAF, 2023; Hochman, 2015; State Comptroller and Ombudsman of Israel, 2018).

Against this background, examining experiences of welfare and health within the context of Eilat provides an opportunity to explore how access to institutional and community-based support shapes opportunities for integration and belonging under conditions of prolonged institutional ambiguity.

From the perspective of Allport’s (1954) intergroup contact theory, limited institutional support weakens one of the central conditions required for meaningful intergroup contact. When access to welfare and health services depends primarily on emergency responses or non-governmental organisations rather than stable institutional engagement, opportunities to build trust, reciprocal relationships, and broader social inclusion may be constrained. Systematic reviews suggest that psychosocial interventions may reduce symptoms of common mental disorders among asylum seekers and refugees, while social-capital-based interventions may improve mental-health and social-capital outcomes; however, both evidence bases remain limited and heterogeneous (Turrini et al., 2019; Villalonga-Olives et al., 2022). Examining welfare and health through both structural and relational perspectives therefore provides important insight into how institutional support facilitates, or constrains, the integration and sense of belonging of Sudanese asylum seekers in Israel.

Recent studies of Eritrean asylum seekers in Tel Aviv provide an important point of comparison for understanding processes of integration and belonging within the Israeli context. Lev Ari and Herscovici (2025) demonstrate that integration is neither uniform nor linear, as asylum seekers may be incorporated into certain sectors of society while remaining excluded from others, a pattern conceptualised as differential exclusion. Alongside the constraints imposed by state policy, relationships with Israeli professionals, employers, and friends may contribute to a stronger sense of belonging and even to feeling “at home.” Herscovici and Lev Ari (2025) further show that the incorporation of asylum seekers in Tel Aviv is shaped by complex interactions among central government, municipal authorities, and civil society organisations. While municipal and civil society initiatives promote community strengthening and provide educational, social, and legal services, national-level policies may simultaneously foster separation and exclusion, resulting in partial integration, or incorporation, rather than full integration. These findings provide an important reference by demonstrating that participation in particular domains of society does not necessarily translate into broader inclusion or secure belonging. The present study shifts the analytical focus from Tel Aviv to Eilat, a peripheral city with distinct geographical, economic, and social characteristics, thereby enabling an examination of how local context shapes the relationship between economic participation, institutional support, social connections, and experiences of belonging.

## 4 The impact of October 7 on Sudanese asylum seekers in Israel

The events of October 7, 2023, and the subsequent war created a new emergency context that intensified existing vulnerabilities among asylum seekers in Israel. Although the interviews analysed in the present study were conducted prior to these events, the post-October 7 period provides an important contextual backdrop for interpreting the study’s findings, as it exposed and intensified structural vulnerabilities that had already characterised the everyday lives of asylum seekers.

While the war affected Israeli society as a whole, asylum seekers experienced its consequences without the protection of a comprehensive social safety net or stable access to welfare and public services. Existing legal uncertainty, limited social rights, and dependence on civil society organisations became even more pronounced during the emergency, reinforcing patterns of insecurity that had already been documented before the war (ASSAF, 2023; State Comptroller and Ombudsman of Israel, 2018; Hotline for Refugees and Migrants, 2021).

According to ASSAF’s activity report for 2023, the war deepened poverty and food insecurity among asylum seekers, displaced some from their homes, and reactivated earlier trauma among people with histories of war, persecution, trafficking, and torture. Access to mental health services remained severely limited, while requests for humanitarian and psychosocial assistance increased sharply (ASSAF, 2024).

The post-October 7 context is particularly relevant to Eilat because of the city’s distinctive economic structure and peripheral location. Approximately 90 per cent of businesses operating in Eilat depend on tourism, leaving the city particularly vulnerable to disruptions in tourism activity during national emergencies (State Comptroller and Ombudsman of Israel, 2025). Following October 7, tens of thousands of evacuees from northern and southern Israel were accommodated in the city, temporarily doubling its population, while the tourism sector and local businesses experienced substantial economic disruption. The State Comptroller concluded that Eilat’s heavy dependence on tourism, together with incomplete implementation of earlier government measures intended to strengthen the city’s economic resilience, left it particularly exposed to the effects of the war (State Comptroller and Ombudsman of Israel, 2025).

For the present study, these developments are especially relevant because Eilat’s tourism-based economy constitutes an important context for understanding the employment and everyday integration of Sudanese asylum seekers. However, because the interviews were conducted before October 7, the study does not provide empirical evidence regarding the direct effects of the war on the Sudanese asylum-seeking community in Eilat. The post-October 7 developments are therefore considered here as an important contextual development rather than as part of the empirical findings.

Rather than creating entirely new patterns of exclusion, the events following October 7 exposed and intensified structural vulnerabilities that had characterised the lives of asylum seekers long before the outbreak of the war. The crisis therefore reinforces the relevance of examining how prolonged institutional ambiguity shapes integration and belonging, while also underscoring how such vulnerabilities may become particularly consequential during periods of national emergency.

## 5 Research objectives and research questions

Specifically, the objectives of this study are as follows:

*O1*: To examine how Sudanese asylum seekers living in Eilat construct, experience, and negotiate belonging under conditions of prolonged institutional ambiguity and the absence of permanent legal status.

*O2*: To examine the mechanisms through which four central domains of integration—employment, housing, education, and welfare and health—enable or constrain social inclusion and belonging among Sudanese asylum seekers in Eilat.

*O3*: To identify and examine gaps between formal policies and institutional practices and the everyday lived experiences of Sudanese asylum seekers in Eilat, and to assess how these gaps shape opportunities for social integration and belonging.

Aligned with these objectives, the study addresses the following research questions:

*RQ1*: How do Sudanese asylum seekers in Eilat experience and narrate belonging under conditions of prolonged institutional ambiguity and the absence of permanent legal status?

*RQ2*: Through what mechanisms do the four domains of integration (employment, housing, education, and welfare and health) enable or constrain social inclusion and belonging?

*RQ3*: What gaps emerge between formal policies and institutional practices and the everyday lived experiences of Sudanese asylum seekers in Eilat, and how do these gaps shape opportunities for social integration and belonging?

## 6 Methodology

### 6.1 Design

This study employed a qualitative interpretive design informed by phenomenological and narrative perspectives to examine how Sudanese asylum seekers living in Eilat experience, interpret, and construct a sense of belonging under conditions of prolonged institutional ambiguity and the absence of permanent legal status. The phenomenological orientation foregrounded participants’ lived experiences and the subjective meanings they attributed to integration, exclusion, and belonging, while the narrative orientation supported examination of how these experiences were described and organised within personal stories and life trajectories.

This design is particularly appropriate because belonging is conceptualised as a dynamic and context-dependent process rather than as a fixed condition or a binary outcome of integration or non-integration. Accordingly, the study focuses on participants’ perspectives and on how their everyday experiences of inclusion and exclusion are shaped within the distinctive social and institutional context of Eilat.

### 6.2 Instruments

The primary data collection instrument was an in-depth semi-structured interview. The interview guide was developed specifically for this study and was not directly adapted from an existing validated instrument. Its development was informed by the research questions and theoretical framework, as well as by the researcher’s sustained professional familiarity with issues affecting the Sudanese asylum-seeking community. The semi-structured format enabled systematic exploration of the study’s central topics while preserving sufficient flexibility for participants to elaborate on personal experiences and narrate their experiences in their own words.

The interview guide comprised 17 open-ended questions organised into three sections. The first section focused on migration history and settlement trajectories and included five questions addressing life in the country of origin, reasons for departure, the migration journey, initial arrival in Israel, and the decision to settle in Eilat. The second section comprised eight questions examining participants’ everyday experiences across the four central domains of integration, employment, education, housing, and welfare and health, as well as their interactions with state and municipal institutions. The third section included four narrative questions exploring experiences of belonging, community involvement, intergroup social ties, encounters with discrimination, and participants’ perceptions of their personal and familial needs.

All interviews were fully documented and transcribed to facilitate systematic analysis and methodological transparency. The complete interview guide is provided in the Supplementary material.

### 6.3 Participants

A non-probabilistic purposive sampling strategy was employed, specifically homogeneous purposive sampling based on predefined eligibility criteria. The sampling strategy was designed to recruit members of the Sudanese asylum-seeking community in Eilat who shared characteristics relevant to the research questions and could provide in-depth accounts of integration and belonging as long-term residents of the city. Recruitment was subsequently facilitated through community-based snowball procedures, enabling access to eligible participants within this hard-to-reach population.

The inclusion criteria were defined in advance. Participants were required to: (1) have lived in Eilat for at least 5 years; (2) be the parent of at least one child; (3) have basic proficiency in Hebrew or English; and (4) participate to some extent in the local labour market. These criteria were intended to ensure that participants had substantial experience of living in Eilat and relevant experience across several of the central domains examined in the study. Exclusion criteria included residence in Eilat for fewer than 5 years or no direct affiliation with the Sudanese asylum-seeking community.

The sample consisted of 30 Sudanese asylum seekers residing in Eilat, aged 27–54 years, with a mean age of 36.77 years (SD = 5.71). Participants had lived in Israel for an average of 14.57 years (SD = 1.86; range = 11–18) and in Eilat for an average of 9.33 years (SD = 1.79; range = 6–12), indicating prolonged residence both in Israel and in the city. The sample comprised 25 men (83.3 per cent) and five women (16.7 per cent). Most participants were married (*n* = 25; 83.3 per cent), four were widowed (13.3 per cent), and one was engaged (3.3 per cent). Detailed demographic characteristics of the sample, including age, gender, family status, and length of residence in Israel and Eilat, are presented in Table 1.

**Table 1:** Participant characteristics.

Variable	Value
Age, M (SD)	36.77 (5.71)
Gender	Male - 25 (83.3 per cent) Female - 5 (16.7 per cent)
Years in Israel, M (SD)	14.57 (1.86)
Years in Eilat, M (SD)	9.33 (1.79)
Family status	Married — 25 (83.3 per cent) Widowed — 4 (13.3 per cent) Engaged — 1 (3.3 per cent)

Age range = 27–54 years; Years in Israel range = 11–18; Years in Eilat range = 6–12. Sample characteristics (*N* = 30).

### 6.4 Procedure

The research procedure followed a systematic and sequential process, with particular attention to ethical considerations and the sensitivity required when conducting research with a population experiencing legal and social vulnerability.

Prior to formal data collection, an initial phase of trust-building and familiarisation with the Sudanese community in Eilat was undertaken. The researcher engaged in voluntary activities at the local community centre, facilitating parental groups addressing intergenerational challenges and processes of cultural adaptation. This involvement provided familiarity with the community context and contributed to establishing conditions of trust and openness prior to data collection. The researcher’s prior engagement with the community was also considered as part of the study’s reflexive approach, given the potential influence of prior familiarity on both data collection and interpretation.

Participant recruitment was initiated through the local community centre, which served as the initial point of contact, and continued through community-based snowball procedures. All potential participants identified through referrals were screened according to the predefined inclusion criteria established within the purposive sampling strategy. Participants received a clear and standardised verbal explanation of the study’s aims, procedures, use of the data, and ethical safeguards. Participation was voluntary and without financial compensation, and participants were informed that they could withdraw from the study at any stage.

Prior to each interview, oral informed consent was obtained. Given participants’ legal vulnerability and the potential reluctance to sign formal documentation, the consent procedure was designed to be accessible, non-coercive, and culturally sensitive. All procedures involving human participants were conducted in accordance with the ethical principles of the Declaration of Helsinki and applicable institutional guidelines (World Medical Association, 2013).

Interviews were conducted face to face in locations chosen by the participants, typically in their homes or at the local community centre, with careful attention to privacy. Each interview lasted approximately 60–90 min and was audio-recorded with the participant’s permission. Data collection took place between January and June 2023. Accordingly, all interviews were conducted prior to the events of October 7, 2023.

Interviews were conducted in Hebrew or English according to each participant’s preference. Each interview was transcribed in full, with attention to preserving the meaning of participants’ accounts as well as relevant linguistic and cultural nuances. Where translation between languages was required for analysis and presentation of the findings, a meaning-oriented approach was used to preserve participants’ intended meanings and the cultural context of their accounts as closely as possible.

To maintain confidentiality, names and identifying information were removed from the transcripts, and participants are identified in the manuscript by pseudonymous initials. Audio recordings and identifiable interview materials were stored in a secure, restricted-access environment accessible only to the lead researcher. De-identified transcripts were shared with the external analyst solely for the purposes of coding and thematic analysis.

### 6.5 Data analysis

Data analysis was conducted using NVivo 12, which was used to organise the transcripts, code the data, group related codes, and examine relationships across categories and themes. The analysis followed a hybrid deductive–inductive thematic approach, allowing theoretically informed concepts to be considered alongside patterns and meanings emerging directly from participants’ accounts. The initial coding and thematic analysis were undertaken with paid assistance from Academia, an external company specialising in academic research support, using de-identified interview transcripts. The lead researcher reviewed the coding and thematic analysis against the full transcripts and worked iteratively with the external analyst in developing and selecting the final themes.

The analytical process proceeded through several systematic stages. First, the transcripts were read repeatedly to develop familiarity with the data and the context of each interview. Meaningful units of text were then identified and assigned initial codes. Inductive coding was used to identify experiences, meanings, and patterns emerging from the empirical material without restricting the analysis to predetermined theoretical categories. In parallel, deductive coding was informed by sensitising concepts derived from Allport’s (1954) intergroup contact theory and Ager and Strang’s (2008) multidimensional integration framework. These theoretical perspectives therefore guided analytical attention without precluding the identification of additional patterns and themes emerging from the data.

Related codes were subsequently grouped into broader categories and preliminary themes. These themes were iteratively reviewed against the full transcripts and across participants to assess their coherence and correspondence with the empirical material. Where necessary, themes were refined, combined, differentiated, or reorganised as the analysis developed. Attention was also given to accounts that diverged from dominant patterns in order to preserve variation within the data and reduce the risk of overgeneralisation.

Cross-domain matrices were subsequently constructed to examine the relationships between Allport’s (1954) conditions for intergroup contact and the integration domains and social connections identified in Ager and Strang’s (2008) framework. These matrices facilitated the identification of convergences, tensions, and relational patterns across employment, education, housing, welfare and health, institutional support, social connections, and experiences of inclusion, exclusion, and belonging.

The final stage involved refining and defining the central themes and relating them back to participants’ accounts and the study’s research questions. Throughout the analytical process, particular attention was given to maintaining a clear connection between the empirical material and its theoretical interpretation. This process provided the analytical foundation for the themes presented in the Results section.

## 7 Results

The results are organised in accordance with the study objectives and the three research questions. First, we examine how participants experience and narrate belonging under conditions of prolonged institutional ambiguity and the absence of permanent legal status (RQ1). We then examine the mechanisms through which the four central domains of integration—employment, housing, education, and welfare and health—enable or constrain social inclusion and belonging (RQ2). Finally, we examine gaps between formal policies and institutional practices and the everyday lived experiences of Sudanese asylum seekers in Eilat, and how these gaps shape opportunities for social integration and belonging (RQ3).

The thematic analysis identified five core themes reflecting different stages and dimensions of participants’ experiences, from displacement and loss, through their initial encounters with Israeli institutions, to their attempts to rebuild stability, social connections, and belonging in Eilat. The themes highlight the interplay between structural and institutional conditions, social relationships, and subjective experiences of inclusion and exclusion. Each theme is presented through an analytical narrative supported by direct quotations from participants, while divergent or contrasting experiences are also included where they emerged from the data. Quotations are accompanied by participants’ pseudonymised identifiers and selected demographic characteristics (gender, age, and/or number of years of residence in Eilat).

### 7.1 Theme one: uprooted roots – lives severed in an instant

For many participants, their narratives of life in Israel began with memories of Sudan. They described their lives before displacement in terms of home, family, community, and belonging, memories that stood in stark contrast to the violence that ultimately forced them to flee. The transition from a familiar sense of belonging to forced displacement was described not as a gradual process, but as an abrupt rupture that fundamentally altered the course of their lives.

L. (Male, 42, 9 years in Eilat) described the destruction of his village: “We heard planes and then explosions […] we saw smoke and fire […] the farm was burned, all the animals died.” K. (Male, 37, 11 years in Eilat) carried with him a sensory memory that remained vivid years later: “I cannot forget the smell of the burned village.” For others, the loss was also deeply personal and familial. A. was forced to bury his parents and brother alone, while L. lost his younger sister during the escape. P. (Male, 42, 9 years in Eilat) described the moment his parents enabled his departure: “My parents gave me all their money […] I have not seen them since”.

The journey itself was similarly described as an experience marked by danger, loss of control, and dependence on others. Some participants travelled through Egypt and reported experiences of violence, detention, and danger along the way. M. (Male, 39, 9 years in Eilat), who was compelled to pay smugglers to cross the Sinai toward Israel, described the absence of alternatives available to him: “It cost me everything I had […] but I knew I had no other choice”.

These accounts show that participants’ experiences of belonging in Eilat did not begin with their arrival in the city. Rather, they were rooted in earlier experiences of losing home, family, community, and security, and in the subsequent need to rebuild stability following forced displacement. In this sense, belonging is not merely a question of integration into a new society, but also of whether and how a sense of home can be reconstructed after an earlier form of belonging has been violently disrupted.

### 7.2 Theme two: a cold reception – from narratives of rescue to a reality of rejection

For many participants, arrival in Israel was initially accompanied by the hope that the danger and uncertainty of their journey had come to an end. This expectation, however, was quickly replaced by uncertainty and a sense of rejection in their encounters with state authorities. A. (Male, 36, 10 years in Eilat) described the abrupt transition between these experiences: “I thought the nightmare was over […] then immigration officers came and took me without explanation to Holot. I did not understand it was a prison”.

Detention in the Holot and Saharonim facilities was described as an experience marked by loss of freedom, time, and meaning. S. (Male, 43, 12 years in Eilat) recalled: “We are imprisoned like criminals, there is nothing to do in there”. For participants, these experiences shaped an early encounter with state institutions in which expectations of protection were replaced by a sense of being treated as individuals to be controlled and restricted.

Release from detention was likewise described as occurring without an institutional support framework to facilitate the transition to independent life. Some participants recounted arriving in South Tel Aviv without housing, guidance, or basic information about where to go or what to do next. T. (Male, 29, 12 years in Eilat), who arrived in Israel as a minor, recalled: “I thought they would tell us where to go, where to sleep. But they simply said: get off the bus”. In these circumstances, more established asylum seekers were often described as the only immediate source of assistance, providing food, temporary accommodation, and initial guidance.

These accounts reveal an early gap between participants’ expectations of finding safety in Israel and their actual experiences of state institutions. Institutional uncertainty therefore did not begin in Eilat, but was already present in participants’ initial encounters with Israel’s reception and control systems. This experience provides an important context for understanding their subsequent move to Eilat, where opportunities for employment, greater stability, and the rebuilding of everyday routines offered possibilities for reconstruction, but did not necessarily transform their uncertain relationship with state institutions or produce a secure sense of belonging.

### 7.3 Theme three: Eilat as a space for reconstruction – between challenges and achievements

For participants, moving to Eilat marked a significant shift from the experiences that had characterised their initial period in Israel. The city’s tourism-based economy and demand for workers in the service and hospitality sectors created opportunities for relatively rapid entry into the labour market. Employment provided not only a source of income but also routine and stability that enabled participants to begin rebuilding their everyday lives. S. (Male, 36) described the difference he experienced between Eilat and Tel Aviv: “Finding work in Eilat was easier, definitely. Because here there are many hotels, many restaurants, many places to work. There are not many people here who want to do these kinds of jobs. So it is easier to find work than in Tel Aviv. There, there are many workers and not as many places like here. Here they are happy that we come to work”.

Employment stability also influenced housing opportunities. Participants described moving from overcrowded and unstable living conditions to more independent family housing, even when this meant living in older neighbourhoods and buildings. B. (Male, 46) explained: “The housing in Eilat is better than in Tel Aviv. Even though we do not live in an area of villas in Eilat. It is a neighbourhood with old houses. But here every family has its own home. We do not live eight or 10 people in one small apartment. It is better here. Because there is work, and it is steady, it is easier to find a home in better condition than what we had in Tel Aviv. Not broken, not ruined, not dirty. An old home, but a good home.” His account illustrates how, for some participants, employment and housing were not experienced as separate domains but as interconnected elements in a gradual process of establishing stability in the city.

Eilat was also described as offering particular advantages for families and children. The city’s relatively small size, perceived safety, and accessibility of educational and community frameworks were viewed by some parents as providing their children with greater independence and support. L. (Female, 42) described: “Here it is a small place. Even though my children go to a school that is not near our home, it is farther away, the teachers and everyone help, really. They have friends, the children can go by themselves and it is not frightening like in Tel Aviv. There are activities that the school provides for them. Sometimes they also get help with their studies. Friends who live in Tel Aviv have more difficulties with their children, with school and also with friends. There are no activities, no help. Here it is better for the children”.

Alongside the opportunities provided by the city, the Sudanese community played a central role in creating stability and belonging. Community networks provided emotional support, mutual assistance, religious and cultural spaces, and a partial substitute for family relationships lost through displacement. H. (Male, 49) expressed this directly: “Without our community here, it would be very difficult. Everyone came from a different place in Sudan, from a different tribe. But here, we are all the same. We help each other, we celebrate holidays together, there is a place of worship, there is a community. It is important. We do not have family. They all died. This is our family.” His account illustrates how belonging to the Sudanese community in Eilat could transcend internal differences originating in Sudan and create a shared space of support and identity.

However, the ability to build a stable life in Eilat was not perceived by participants as equivalent to full belonging within Israeli society. Positive relationships with Israelis did exist, particularly in the workplace, but these relationships often remained within the boundaries of cordial and functional interaction rather than developing into close social ties. L. (Female, 42) described this boundary sharply: “There is work, there is a home, there is school for the children. The Israelis at work treat me well. They are always nice, always asking, checking how I am. My boss too, he is a good man who always helps. But they are not really friends like friends from the community. We are not like the Israelis. I do not think we ever will be. We are the Sudanese and they are the Israelis. Even if they treat us well”.

Taken together, these accounts portray Eilat as a space in which participants were able to rebuild everyday life through employment, more stable housing, opportunities for their children, and supportive community networks. At the same time, they reveal a clear boundary between participation in the life of the city and deeper social belonging. Participants could work alongside Israelis, establish homes in the city, and raise their children there for years, while continuing to perceive themselves as a distinct group. This tension between functional stability and limited social belonging emerges as a central feature of participants’ experiences in Eilat and provides the basis for the question explored in the following theme: whether the lives established in the city are experienced as new roots or as an existence that remains temporary and conditional.

### 7.4 Theme four: new roots or a temporary existence?

The fourth theme reveals one of the central tensions in participants’ experiences of belonging: the gap between lives established over many years in Eilat and the absence of a secure future and full sense of belonging in Israel. As the previous theme demonstrated, employment, housing, and education enabled participants to build relatively stable everyday lives in the city. Yet this stability did not necessarily transform Eilat into “home” in an emotional and social sense. For some participants, home remained associated with the places, relationships, and lives they had lost in Sudan. S. (Male, 38) described this tension: “Eilat is good. Really. Much better than Tel Aviv. It was difficult there. Here it is fine. But I miss Sudan all the time. What we had there. The village, the family, the friends. There I feel at home. Here it is not really a real home”.

The tension between integration and belonging became even more complex when participants spoke about their children. For parents whose children were born and raised in Israel, spoke Hebrew, and participated in local educational and social settings, the gap between everyday belonging and the absence of formal recognition was perceived as a significant concern for the future. D. (Male, 39) explained: “My children were born in Israel. They speak almost only Hebrew and English, not our language at home. But what will happen when they grow up and finish high school? No army, no university, nothing. They are kind of Israeli, but not really. They only feel that way.” His account illustrates how a sense of belonging may develop culturally, linguistically, and socially while the future remains perceived as uncertain because of the absence of secure status and rights.

For some participants, this uncertainty directed their attention beyond Israel. Other countries were imagined not simply as possible migration destinations, but as places where integration might be accompanied by rights and the possibility of building a more secure future for the next generation. D. (Male, 39) described this through the experience of friends who had moved to Canada: “I have friends who left Israel and went to Canada. It is better there. They have rights, work, a good school for the children, a big house. I also think I will leave eventually. What are my children supposed to do here without rights? Be like me? Work in a hard job?” Importantly, the possibility of leaving was not presented as a consequence of an inability to establish a life in Eilat. Rather, it emerged after years of employment, settlement, and raising children in the city.

Taken together, these accounts point to a form of conditional belonging: participants participate in the life of the city, work, establish households, and raise their children there, yet their sense of belonging remains constrained by uncertainty regarding their status and future. For the second generation, this tension is particularly pronounced: children may experience themselves as Israeli in linguistic, social, and cultural terms, while their parents question whether this belonging will be formally recognised or translated into future opportunities. The belonging emerging from these accounts is therefore neither simply an absence of belonging nor merely a temporary condition. Rather, it is a form of belonging constructed through everyday life yet remaining insecure and conditional, a situation in which individuals can build a life in a place without knowing whether that place can become a permanent and recognised home.

### 7.5 Theme five: embrace and distinction – the internal dynamics of the community

The fifth theme focuses on the dual role of the Sudanese community in Eilat: as a central source of support, cultural continuity, and belonging, while simultaneously constituting a distinct social space within which many of the participants’ most meaningful relationships are maintained. In the absence of extended family networks and sufficient formal support structures, the community performs functions that might otherwise be provided by family or social institutions.

Mutual assistance was described as an integral part of everyday community life rather than merely a response to emergencies. S. (Male, 42) explained: “Here, people in the community help each other. If a child is sick, they help; if a woman gives birth, they bring her food; if an elderly person is sick, people come to take care of him. It is like a family.” This account illustrates how the community functions as an informal network of care and support, providing practical and emotional security and partially compensating for the absence of family ties and other support mechanisms.

Alongside everyday support, the community also serves as a space for maintaining and transmitting cultural identity to the next generation. O. (Male, 37) described: “We have a club, a place of worship. We meet during holidays, we pray, we have meals together. Our children learn once a week about the history of the Sudanese people, they learn the language. Not all the children want to […] but everything is there.” His account also points to an intergenerational complexity: while adults create frameworks intended to preserve language, history, and tradition, not all children demonstrate the same desire to participate in them. The community therefore becomes not only a space for cultural preservation but also a setting in which the meaning of Sudanese identity is negotiated among a generation growing up in Israel.

At the same time, the strength of relationships within the community existed alongside the relative weakness of deeper social ties with Israelis. O. (Male, 39) distinguished between everyday interaction and close friendship: “Real friends are only other Sudanese. There are almost no Israeli friends. Maybe at work we talk and laugh, but that is it. It does not continue beyond that. Because only a Sudanese person understands all the ways of behaving and the things that are important to another Sudanese person.” His account highlights how frequent contact with Israelis, particularly in the workplace, does not necessarily develop into relationships extending beyond the settings in which such encounters occur. Shared cultural understandings and experiences make the Sudanese community a space in which participants can experience forms of intimacy and mutual recognition that are less readily available outside it.

Taken together, these accounts reveal the dual character of the Sudanese community in Eilat as both buffer and boundary. On the one hand, it functions as a vital protective network, providing care, mutual assistance, cultural continuity, and a sense of family and belonging. On the other hand, the concentration of participants’ most meaningful social relationships within the community may sustain a social boundary between the Sudanese community and the wider Israeli society. Importantly, the findings do not necessarily indicate that the community itself prevents broader integration. Rather, they suggest that, under conditions in which relationships with Israelis often remain functional and confined to settings such as the workplace, the community provides forms of deeper social connection that are less frequently developed outside it.

This duality further illuminates the pattern of conditional belonging identified in the preceding theme. Participants are not socially or economically isolated from the local society, yet much of their sense of home, recognition, and social intimacy is constructed within the Sudanese community. The community therefore operates simultaneously as a resource that enables participants to establish and sustain everyday life in Eilat and as a social boundary reflecting the limitations of their broader belonging within Israeli society.

### 7.6 Summary of findings

The findings portray the experiences of Sudanese asylum seekers in Eilat as a trajectory extending from displacement and loss to the reconstruction of stability and belonging. Experiences of violence and forced displacement in Sudan, followed by participants’ initial encounters with Israeli state institutions, formed the background against which they attempted to rebuild their lives. For many participants, moving to Eilat marked a significant shift. The city’s tourism-based economy provided relatively accessible employment opportunities, employment stability contributed to improved housing conditions, and the relatively small scale of the city was perceived by some parents as offering a safer and more supportive environment for their children.

At the same time, the findings demonstrate that functional stability did not necessarily translate into social or civic belonging. Even after years of residence in Eilat, employment alongside Israelis, and raising children in the city, many participants described limited social ties with the wider Israeli society and continuing uncertainty regarding their status and future. This tension was particularly evident in parents’ accounts of children who were born and raised in Israel and identified with the local language and everyday life, while their future was nevertheless perceived by their parents as uncertain.

Within this context, the Sudanese community in Eilat played a central role as a source of practical and emotional support, cultural continuity, and a sense of family. Community networks provided assistance during illness, childbirth, and other everyday needs, while also creating spaces for the maintenance and transmission of language, traditions, and cultural identity to the next generation. At the same time, the concentration of participants’ deepest social relationships within the community highlighted the continuing boundary between participation in the life of the city and broader social belonging. The community thus operated as both buffer and boundary: a vital source of security and belonging, while simultaneously constituting a distinct social space that reflected the limitations of participants’ relationships with the wider Israeli society.

Taken together, the five themes reveal a pattern of conditional belonging. Eilat enabled participants to reconstruct central elements of everyday life, to work, establish more stable housing, raise children, and develop supportive networks, yet these achievements coexisted with institutional uncertainty and the absence of a secure sense of full belonging. The findings therefore point to a central distinction between functional integration and secure belonging: individuals may participate continuously in the economic and social life of a place, and may build their lives there over many years, without such participation necessarily translating into recognition, security regarding the future, or a sense that the place constitutes a permanent and fully recognised home.

## 8 Discussion

The findings of this study demonstrate that the experiences of Sudanese asylum seekers in Eilat cannot be adequately understood through the presence or absence of integration alone. Rather, they reveal a distinction between functional integration and secure belonging. Participants had established relatively stable lives through employment, housing, their children’s participation in education, and strong community networks, yet these forms of everyday participation coexisted with continuing legal and institutional uncertainty and limited social recognition. Eilat therefore emerges as a context in which reconstruction and participation are possible without necessarily producing a secure or fully recognised sense of belonging.

### 8.1 Functional integration and conditional belonging

The findings addressing RQ1 indicate that belonging under conditions of prolonged legal and administrative ambiguity is neither simply absent nor fully achieved. Participants had lived in Israel and Eilat for many years, worked, established households, raised children, and developed meaningful attachments to the city. These experiences demonstrate substantial participation in several of the domains identified by Ager and Strang (2008). Nevertheless, such participation did not consistently translate into a secure sense of belonging.

This distinction is particularly visible in participants’ accounts of Eilat as a place in which they had successfully reconstructed everyday life while remaining uncertain about whether Israel could become a permanent and recognised home. Participants could describe Eilat positively, particularly in comparison with their earlier experiences in Tel Aviv, while simultaneously maintaining emotional attachments to Sudan and expressing uncertainty regarding their future in Israel. Belonging therefore emerged as a negotiated and contingent experience rather than as a linear outcome of prolonged residence or socioeconomic participation.

The experiences of participants’ children further illuminate this tension. Children born or raised in Israel may speak Hebrew, participate in Israeli schools, develop local friendships, and experience Israeli society as the primary social environment they know. Yet parents remained concerned that this everyday and cultural embeddedness would not necessarily translate into secure recognition or future opportunities. The resulting gap between lived participation and anticipated recognition demonstrates what this study conceptualises as conditional belonging: belonging constructed through everyday life and social participation but remaining contingent upon institutional conditions that individuals themselves cannot fully control.

This finding extends the analytical distinction between structural participation and social connections within Ager and Strang’s (2008) framework. Participation in employment, housing, and education may provide essential foundations for integration, but the findings from Eilat suggest that these achievements are insufficient on their own to produce secure belonging when institutional recognition remains uncertain. Belonging must therefore be understood not merely as an outcome of accumulated participation across domains, but as a relational and institutional process through which participation becomes, or fails to become, recognised membership.

To clarify how this process operates across the empirical findings, Table 2 integrates the three research questions with Ager and Strang’s (2008) integration framework and Allport’s (1954) contact theory. The table illustrates how participation within central life domains may generate functional stability while producing different outcomes depending on the presence or absence of equal status, cooperation, shared goals, and institutional support.

**Table 2:** Integration of empirical findings, research questions, and theoretical interpretation through Ager and Strang’s (2008) integration framework and Allport’s (1954) contact theory.

Research question (RQ)	Key findings	Theoretical contribution	Implications for policy/practice
RQ1: How do Sudanese asylum seekers in Eilat experience and narrate belonging under conditions of prolonged institutional ambiguity and the absence of permanent legal status?	Participants developed everyday stability and attachments to Eilat while continuing to experience uncertainty regarding status, recognition, and future prospects. Belonging was therefore neither absent nor secure, but conditional, particularly for parents concerned about the future of children born and raised in Israel.	Extends the analysis of integration beyond participation in structural domains by distinguishing functional integration from secure belonging. It illustrates how sustained participation may coexist with limited recognition and institutional uncertainty.	Greater legal clarity, institutional recognition, and pathways that enable long-term residents and their children to translate everyday participation into more secure forms of social belonging.
RQ2: Through what mechanisms do the four domains of integration (employment, housing, education, and welfare and health) enable or constrain social inclusion and belonging?	Eilat facilitated functional stability through relatively accessible employment, improved family housing, and educational opportunities for children. However, participation across these domains did not consistently generate deeper social inclusion, while access to welfare and health support remained limited and fragmented.	Shows how Ager and Strang’s (2008) integration domains can function as settings for intergroup contact. Read through Allport (1954), participation may be associated with stronger integrative outcomes when contact is accompanied by equal status, cooperation, shared goals, and institutional support.	Employment and service access should be accompanied by cultural and linguistic mediation, sustained institutional support, and opportunities for relationships extending beyond functional encounters.
RQ3: What gaps emerge between formal policies and institutional practices and the everyday lived experiences of Sudanese asylum seekers in Eilat, and how do these gaps shape opportunities for social integration and belonging?	Local opportunities enabled participants to establish relatively stable everyday lives, but national-level legal and institutional uncertainty remained unresolved. Positive everyday encounters with Israelis, particularly through employment, frequently remained functional rather than developing into close social relationships. Sudanese community networks provided essential support, cultural continuity, and social intimacy where broader bridging ties remained limited.	Illustrates the potential limits of local integration under national institutional ambiguity: favourable local conditions may facilitate reconstruction and functional participation without overcoming structural uncertainty. The findings suggest that the Sudanese community may operate as both buffer and boundary, providing belonging while reflecting the limited development of deeper bridging ties.	Local integration initiatives should be supported by coherent national policy. Community-based support should be strengthened alongside opportunities for sustained bridging contact, rather than treated as a substitute for institutional responsibility.

### 8.2 Eilat as a distinctive peripheral integration context

The findings addressing RQ2 demonstrate the importance of place in shaping integration trajectories. Eilat was not merely the geographical setting in which participants happened to reside; its particular economic, spatial, and social characteristics influenced the opportunities available to them. This is especially important given the predominance of research on African asylum seekers in Israel that has focused on Tel Aviv and the central region. Examining Eilat therefore provides insight into how integration may develop differently within a peripheral tourism-based city.

Participants consistently described employment as one of Eilat’s principal advantages. The concentration of hotels, restaurants, and service-sector employment created relatively accessible opportunities for labour-market participation. Compared with participants’ experiences and perceptions of Tel Aviv, Eilat was described as a setting in which employment could be obtained more readily and maintained with greater continuity. This finding is consistent with research identifying the importance of Eilat’s tourism economy and labour-market structure in shaping the incorporation of asylum seekers into the city (Barak-Bianco and Raijman, 2025).

Importantly, the benefits of employment extended into other domains. Greater employment stability enabled some participants to secure better and less overcrowded housing, while parents described Eilat’s relatively small scale as providing advantages for their children, including perceived safety, educational support, extracurricular activities, and greater independence. These findings suggest that integration domains are interconnected: improvement in employment may facilitate housing stability and family well-being rather than operating as an isolated indicator of economic incorporation.

Yet Eilat’s capacity to facilitate everyday reconstruction had clear limits. Participants’ accounts demonstrated that frequent interaction with Israelis, particularly through employment, did not necessarily develop into close social relationships. Cordial workplace relations could coexist with strong perceptions of social distinction between Sudanese asylum seekers and Israelis. Eilat therefore presents an analytically important case precisely because relatively favourable local conditions did not eliminate broader boundaries of belonging. The city enabled functional integration, but the depth of social inclusion remained uneven.

This finding also qualifies any simple contrast between Eilat and Tel Aviv. The present study does not constitute a systematic comparative analysis between the two cities, and participants’ references to Tel Aviv should therefore be understood as experiential points of comparison rather than evidence of city-level causal differences. Nevertheless, their accounts demonstrate that local urban conditions matter: Eilat offered opportunities for employment, housing stability, and family life that participants themselves perceived as more accessible, while national-level uncertainty remained unchanged.

### 8.3 Integration domains as sites of intergroup contact

A central theoretical contribution of the study lies in bringing Ager and Strang’s (2008) integration framework into dialogue with Allport’s (1954) intergroup contact theory. Rather than treating these perspectives as parallel explanatory frameworks, the present analysis conceptualises the domains of employment, housing, education, and welfare and health as settings in which intergroup contact occurs. Ager and Strang therefore help identify where integration is taking place, while Allport provides a means of examining the conditions under which interaction within those domains may develop into broader social inclusion.

Employment provides the clearest illustration. Participants’ employment in Eilat generated repeated encounters with Israeli employers, colleagues, customers, and other members of the host society. Such encounters provided opportunities for cooperation and, in some cases, supportive interpersonal relationships. Nevertheless, participants’ accounts also showed that these relationships frequently remained confined to the workplace. Contact therefore occurred, but it did not consistently generalise into friendship or broader social belonging.

Viewed through Allport’s (1954) framework, the findings suggest that frequency of contact alone is insufficient. Positive intergroup contact is more likely to produce broader relational change when it occurs under conditions of equal status, cooperation, common goals, and institutional support. In the present study, cooperation was often evident in everyday working relationships, but equal status and institutional security remained considerably weaker. This interpretation is consistent with subsequent meta-analytic research demonstrating that intergroup contact generally reduces prejudice while also highlighting the importance of the conditions under which contact occurs (Pettigrew and Tropp, 2006).

Similar dynamics were visible across other domains. Educational participation provided children with sustained exposure to Israeli institutions and peers, while housing established participants within local neighbourhoods and welfare and health services generated encounters with institutional actors. Yet access to these domains varied in quality and stability, and participation did not necessarily provide recognition on equal terms. The findings therefore suggest that structural participation and relational inclusion should not be treated as interchangeable outcomes.

This theoretical integration helps explain the central empirical paradox of the study: participants could be integrated into the everyday functioning of Eilat without experiencing themselves as fully included within the society to which that city belongs. Integration domains provided opportunities for participation and contact, but where equal status and stable institutional support were limited, these encounters were less likely to develop into secure social belonging.

### 8.4 Community as buffer and boundary

The Sudanese community emerged as one of the most important sources of belonging in participants’ accounts. Community networks provided practical assistance during illness, childbirth, and periods of difficulty, while religious, cultural, and educational activities enabled the preservation and transmission of Sudanese identity. For participants who had lost or become separated from family members through displacement, the community could effectively assume familial functions.

These findings are consistent with Ager and Strang’s (2008) distinction between social bonds, bridges, and links. Strong co-ethnic bonds provided resources, recognition, cultural continuity, and emotional security. Rather than interpreting these networks merely as evidence of separation from the host society, the present findings demonstrate their adaptive importance under conditions in which formal institutional support and deeper social relationships outside the community remained limited.

At the same time, participants distinguished sharply between relationships within the Sudanese community and relationships with Israelis. Interaction with Israelis could be positive and frequent, particularly at work, while close friendship remained concentrated largely within the Sudanese community. The community can therefore be understood as both buffer and boundary: a buffer because it mitigates the consequences of displacement, institutional uncertainty, and limited external support; and a boundary because the concentration of intimate social relationships within the community reflects the limited development of bridging relationships with the wider host society.

Importantly, these findings do not establish that strong community ties themselves cause limited integration. Such an interpretation would overlook the institutional and relational context within which these networks developed. An alternative interpretation supported by participants’ accounts is that strong bonding ties become particularly important precisely because relationships outside the community often remain functional rather than intimate. The relationship between bonding and bridging should therefore be understood as contextual and potentially reciprocal rather than as a simple trade-off.

### 8.5 Institutional ambiguity and the limits of local integration

The findings addressing RQ3 demonstrate a fundamental tension between locally available opportunities and nationally determined institutional conditions. Eilat could provide employment, improved housing opportunities, educational participation, and a relatively stable environment for family life. What the city could not provide was resolution of the legal and institutional uncertainty governing asylum seekers’ long-term presence in Israel.

This distinction is central to understanding institutional ambiguity in the present study. The concept refers not simply to uncertainty in an abstract sense, but to a condition in which individuals may participate continuously in the economic and social life of the host society while lacking stable institutional recognition of their long-term membership. Israel’s temporary protection arrangements have prevented the removal of many Sudanese and other African asylum seekers while not providing the stable status and comprehensive social rights associated with recognised long-term membership (State Comptroller and Ombudsman of Israel, 2018). The result is a structural discrepancy between prolonged presence and uncertain recognition.

The findings from Eilat demonstrate how this national-level ambiguity is experienced locally. Participants could achieve forms of stability that would ordinarily be interpreted as indicators of successful integration: continuous employment, independent housing, children embedded in local schools, and long-term residence, while remaining uncertain about their future. Local integration therefore reached a structural ceiling: municipal and community conditions could facilitate everyday reconstruction, but they could not independently resolve the institutional conditions determining status and long-term security.

Civil society and community organisations played an important role within this gap by providing forms of assistance that were unavailable or insufficient through formal systems. Their importance should not be interpreted as evidence that such organisations create dependency. Rather, participants’ reliance on community and non-governmental support reflects the limited reach of institutional provision. This distinction shifts analytical attention from the behaviour of asylum seekers or community organisations to the broader distribution of institutional responsibility.

The Eilat case therefore contributes to the wider literature by demonstrating that favourable local conditions and sustained everyday participation do not necessarily overcome national-level institutional ambiguity. A peripheral city may provide meaningful opportunities for reconstruction and repeated contact with the host society while remaining unable to convert those opportunities into secure membership. Conditional belonging emerges from precisely this disjunction: participants have established lives that are socially and materially rooted in place, while the institutional foundations necessary to make that belonging secure remain uncertain.

Taken together, the findings suggest that integration should not be evaluated solely through participation in employment, housing, education, or other measurable domains. The Eilat case demonstrates that functional integration can coexist with limited bridging ties and institutional uncertainty over prolonged periods. Ager and Strang’s (2008) framework identifies the domains through which everyday integration develops, while Allport’s (1954) theory helps explain why participation and contact within those domains do not automatically generalise into broader social inclusion. The combination of these perspectives reveals the central contribution of the study: integration can become locally functional while belonging remains institutionally conditional.

## 9 Implications of the study

The findings have both theoretical and practical implications for understanding refugee integration under conditions of prolonged institutional ambiguity. Theoretically, the study demonstrates the value of bringing Ager and Strang’s (2008) multidimensional integration framework into dialogue with Allport’s (1954) intergroup contact theory. By conceptualising employment, housing, education, and welfare/health not only as domains of integration but also as settings in which intergroup contact occurs, the study helps explain why sustained participation in the everyday life of a host society does not necessarily develop into broader social inclusion. The findings from Eilat indicate that contact may be frequent and cooperative, particularly in employment, while remaining largely functional when equal status and stable institutional support are limited.

The study further contributes to the conceptualisation of conditional belonging by demonstrating that functional integration and secure belonging should not be treated as equivalent outcomes. Participants were able to establish employment, housing, family routines, and long-term attachments to Eilat while continuing to experience uncertainty regarding recognition, status, and future prospects. This distinction highlights the importance of examining not only whether asylum seekers participate in central domains of society, but also whether such participation is accompanied by the institutional and relational conditions that enable individuals to experience themselves as recognised members of the society in which they live.

The findings also demonstrate the analytical importance of place. Eilat’s tourism-based economy, relatively accessible employment opportunities, and small-city environment facilitated forms of everyday reconstruction that participants often perceived as more favourable than their previous experiences in Tel Aviv. At the same time, these local advantages could not resolve national-level legal and institutional uncertainty. The study therefore suggests that local contexts can substantially shape the conditions of integration while remaining constrained by policies determined at the national level.

From a practical perspective, the findings indicate that integration policies should extend beyond facilitating access to employment or other individual domains. Employment stability was associated with improvements in housing and family life, but participation alone did not consistently generate deeper social belonging. Policies should therefore combine access to employment, housing, education, welfare, and healthcare with linguistic and cultural mediation, sustained institutional support, and opportunities for meaningful interaction between asylum seekers and members of the wider host society.

The findings also underscore the importance of recognising and supporting community-based networks without treating them as substitutes for institutional responsibility. The Sudanese community in Eilat provides essential emotional, practical, and cultural resources and functions as an important source of resilience and belonging. Initiatives aimed at strengthening bridging relationships with the wider society should therefore complement, rather than weaken, these existing community bonds.

Finally, the study points to the need for greater coordination between national government institutions, local authorities, educational and welfare services, and civil society organisations. Greater clarity and consistency in institutional arrangements, together with culturally responsive service provision and sustained opportunities for equal-status interaction, may help narrow the gap between functional participation and secure belonging. The Eilat case suggests that meaningful local integration is possible, but its capacity to develop into durable social inclusion remains limited when the broader institutional conditions governing asylum seekers’ lives remain uncertain.

## 10 Limitations

Several limitations should be considered when interpreting the findings of this study. First, the research was conducted in a single peripheral city and involved a purposively selected sample of 30 Sudanese asylum seekers. The findings are therefore context-specific and are not intended to be statistically generalisable to the broader population of Sudanese asylum seekers in Israel or to refugee populations in other settings. Nevertheless, the qualitative design provides an in-depth, contextually grounded account of participants’ experiences and may offer analytical insights relevant to other settings characterised by prolonged legal uncertainty and uneven opportunities for integration.

Second, the sample was predominantly male, comprising 25 men and five women. Although women’s accounts were included in the analysis, their experiences are necessarily less extensively represented. Gender may shape experiences of employment, family responsibilities, community participation, access to services, and belonging in ways that cannot be fully examined within the present sample. Future research should therefore include more gender-balanced samples and examine gendered integration trajectories in greater depth.

Third, the inclusion criteria required participants to have lived in Eilat for at least 5 years, to be parents of at least one child, to have basic proficiency in Hebrew or English, and to be integrated to some extent into the local labour market. While these criteria were appropriate to the research questions and enabled the study to focus on long-term residents with experience across multiple integration domains, they may have resulted in the underrepresentation of individuals experiencing greater social isolation, unemployment, linguistic barriers, or residential instability. The findings may therefore reflect the experiences of comparatively established members of the Sudanese community more strongly than those living under more precarious conditions.

Fourth, recruitment through community-based snowball sampling may have introduced network-related selection effects. Participants recruited through existing community relationships may share experiences, resources, or perspectives that differ from those of Sudanese asylum seekers who are less connected to community networks. This consideration is particularly relevant to the findings concerning the Sudanese community as a source of support, cultural continuity, and belonging.

Fifth, the study relies partly on participants’ retrospective narratives of forced displacement, arrival in Israel, and earlier stages of settlement. Accounts of events that occurred many years before the interviews may have been shaped by memory, subsequent experiences, and participants’ present interpretations of their past. Within a phenomenological–narrative design, however, these retrospective interpretations remain analytically relevant because the study is concerned with how participants understand and construct meaning around their experiences over time.

Finally, all interviews were conducted between January and June 2023 and therefore preceded the events of October 7, 2023 and the subsequent war. The study cannot consequently make empirical claims regarding how these events altered participants’ experiences or perceptions. The discussion of October 7 elsewhere in the manuscript is therefore presented as subsequent contextual evidence rather than as part of the interview findings. Future longitudinal research could examine whether and how the post-October 7 context has affected employment, institutional access, community relations, and experiences of belonging among Sudanese asylum seekers in Eilat.

Taken together, these limitations define the scope within which the findings should be interpreted. The study provides a contextually grounded account of how long-term Sudanese residents of Eilat experience functional integration, institutional ambiguity, community support, and conditional belonging. Future research across different Israeli cities, more diverse participant groups, and changing institutional and political contexts could further examine which of the patterns identified here are specific to Eilat and which extend beyond this particular urban setting.

## 11 Future research

The findings of this study suggest several directions for future research. First, comparative research across different Israeli cities is needed to examine the extent to which the patterns identified in Eilat are specific to its peripheral, tourism-based urban context. Comparative studies involving cities such as Tel Aviv, Ashdod, and Jerusalem could examine how differences in labour-market structures, housing conditions, municipal services, community networks, and opportunities for intergroup contact shape trajectories of functional integration and belonging. Such research would help distinguish processes that are specific to Eilat from those associated with the broader national institutional context.

Second, greater attention should be directed toward the experiences of the second generation—children and young people who were born or raised in Israel. The findings reveal a particularly important tension between children’s linguistic, educational, and social embeddedness in Israeli society and parental uncertainty regarding their future status and opportunities. Longitudinal research following young people through the transition from school to adulthood could examine how identity, belonging, educational aspirations, employment opportunities, and future orientation develop under conditions of prolonged legal and institutional uncertainty.

Third, future research should adopt longitudinal designs to examine the relationship between functional integration and secure belonging over time. The present study demonstrates that long-term participation in employment, housing, education, and community life does not necessarily translate into secure belonging. Following individuals and families over extended periods could establish whether functional stability eventually facilitates deeper social and relational inclusion, or whether belonging remains conditional in the absence of changes in institutional recognition and legal security.

Fourth, multi-actor research involving asylum seekers, local authorities, schools, employers, welfare and healthcare professionals, community organisations, and NGOs could provide a more comprehensive understanding of how integration is produced across institutional settings. Such research could identify where coordination succeeds or breaks down and examine how local actors respond to gaps between national policy and everyday needs. This would be particularly valuable for understanding the extent to which local initiatives can mitigate, but not necessarily overcome, national-level institutional ambiguity.

Fifth, qualitative research examining the perspectives of members of the host society is needed to complement the accounts presented here. Studies involving employers, colleagues, educators, neighbours, and other local residents could explore why repeated everyday contact sometimes develops into supportive relationships while at other times remains primarily functional. Such research would allow more direct examination of Allport’s (1954) conditions of equal status, common goals, cooperation, and institutional support, and of the circumstances under which contact extends beyond specific settings into broader social relationships (Pettigrew and Tropp, 2006).

Finally, because the interviews were completed before October 7, 2023, future research should examine the experiences of Sudanese asylum seekers in Eilat in the post-October 7 context. Longitudinal or follow-up studies could investigate changes in employment and economic security, access to welfare and health services, relations with local residents and institutions, community support, and perceptions of belonging. Such research would also make it possible to assess whether periods of national crisis alter existing boundaries between asylum seekers and the host society or reinforce patterns of institutional uncertainty identified in the present study.

Together, these directions would enable future research to move beyond the question of whether asylum seekers are integrated and toward a more differentiated examination of where, how, and under what institutional and relational conditions functional participation develops into secure belonging.

## 12 Conclusion

This study examined how long-term Sudanese asylum seekers in Eilat experience and negotiate belonging under conditions of prolonged legal and institutional ambiguity. The findings demonstrate that integration and belonging should not be treated as equivalent outcomes. Participants had established substantial elements of everyday life in Eilat through employment, housing, their children’s participation in education, and strong community networks. Yet these forms of functional integration did not consistently translate into secure social recognition or confidence regarding their long-term future in Israel.

Eilat emerged as an analytically significant setting for understanding this distinction. Its tourism-based economy provided relatively accessible employment opportunities, which in turn supported greater housing stability and the reconstruction of family routines. Participants also described advantages associated with the city’s relatively small scale, particularly for children and families. These findings demonstrate that peripheral urban contexts can generate meaningful local opportunities for reconstruction even in the absence of a comprehensive national integration framework. At the same time, the Eilat case reveals the limits of what favourable local conditions can achieve: sustained economic participation and everyday stability cannot independently resolve legal uncertainty or guarantee broader social belonging.

Across the four domains examined, employment, housing, education, and welfare and health, the findings show that participation was uneven and interconnected. Improvements in one domain could facilitate stability in others, yet access and participation alone were insufficient to produce deeper inclusion. Read together, Ager and Strang’s (2008) integration framework and Allport’s (1954) intergroup contact theory help explain this pattern. The former identifies the domains through which everyday integration develops, while the latter draws attention to the conditions under which interactions within these domains may extend beyond functional encounters. In Eilat, repeated contact with Israelis, particularly through employment, often involved cooperation and positive everyday interaction, but did not consistently develop into close social relationships where equal status and sustained institutional support remained limited.

The Sudanese community was central to this process. Community networks provided practical assistance, emotional security, cultural continuity, and a sense of family and belonging. Rather than viewing strong co-ethnic ties simply as an obstacle to integration, the findings suggest that they should be understood in relation to the broader institutional and relational environment. The community operated as both buffer and boundary: it mitigated the effects of displacement and limited formal support while also constituting the principal space in which many participants experienced deep social recognition and intimacy.

The central contribution of the study is therefore the identification of conditional belonging as a condition in which individuals may become socially, economically, and emotionally rooted in a place while the security and recognition of that belonging remain uncertain. This was particularly visible in participants’ concerns regarding children born or raised in Israel, whose linguistic, educational, and social embeddedness did not necessarily reassure parents that their future membership and opportunities would be secure. The findings thus demonstrate that long-term residence and functional participation can produce meaningful attachment without necessarily producing secure belonging.

From a policy perspective, the findings indicate that facilitating employment or access to individual services, although essential, is insufficient as an integration strategy. Greater coordination between national institutions, local authorities, educational and welfare services, and civil society organisations is required, alongside linguistic and cultural mediation and opportunities for sustained, meaningful intergroup relationships. Community-based networks should be recognised and supported as important resources while not being expected to substitute for institutional responsibility.

As a qualitative study conducted in a single peripheral city, the findings are not intended to be statistically generalisable. Their contribution lies instead in identifying processes through which local opportunities, institutional uncertainty, intergroup contact, and community networks interact over time. Eilat demonstrates a broader theoretical point with potential relevance beyond the Israeli context: a host society may enable people to build a life without providing the conditions that allow them to experience that life as securely and fully theirs. In this sense, the distinction between functional integration and conditional belonging provides a framework for understanding how prolonged participation can coexist with enduring uncertainty in contexts characterised by temporary status and incomplete institutional inclusion.

## Data Availability

The datasets presented in this article are not publicly available due to the sensitive nature of the qualitative interview materials and concerns regarding participant anonymity. Requests to access appropriately anonymised materials should be directed to the corresponding author and will be considered subject to applicable ethical and confidentiality restrictions.
