# Supplementary material for: Conditional belonging under institutional ambiguity: narratives of Sudanese asylum seekers in Eilat, Israel
Source: Front Sociol. 2026 Oct 12;11:1943355. doi: 10.3389/fsoc.2026.1943355 (PMC13696253; doi:10.3389/fsoc.2026.1943355)
Supplement: Supplementary file 1 [file Supplementary_file_1.docx]

Supplementary Material

# 1 Supplementary Data

**Interview Guide**

**Opening and Ethical Clarification**

Introduction of the interviewer, research topic, and objectives.
Brief background information (age, marital status, country of origin, years in Israel).
Clarification of confidentiality and anonymity.

**Section I – Migration History and Settlement Trajectory**

1. Can you describe your life in Sudan before leaving, including childhood and family experiences?
2. What events or circumstances led you to decide to leave Sudan?
3. How did you experience your journey to Israel?
4. Which city did you first arrive in, and what were your initial experiences there?
5. Why did you choose to settle in Eilat?

**Section II – Everyday Integration Across Life Domains**

6. How would you describe your overall integration experience in Eilat?
7. Tell me about your experiences in the labour market and in your current	workplace.
8. Do you feel treated as an equal at work? Please describe specific situations.
9. How have your children been integrated into the education system?
10. What challenges or gaps do you perceive in the educational support your children receive?
11. Tell me about your housing situation and your living environment.
12. How would you describe your access to welfare and health services?
13. Have you encountered difficulties in dealing with municipal or state institutions (such as City Hall, Ministry of Interior, welfare services, or education authorities)? Please explain.

**Section III – Belonging, Community, and Institutional Relations**

14. Tell me about your involvement in the Sudanese community in Eilat and the support it provides.
15. Have you formed social connections outside your community? How do these relationships affect your daily life?
16. Can you describe an experience in which you felt discriminated against because of your origin?
17. Overall, what do you feel you lack today, as an individual, as a family member, and as a resident in Israel?
